# MCM - 2 and Ki - 67 as proliferation markers in renal cell carcinoma: A quantitative and semi - quantitative analysis

**DOI:** 10.1590/S1677-5538.IBJU.2015.0388

**Published:** 2016

**Authors:** Muhammad Zain Mehdi, Abdul Hanan Nagi, Nadia Naseem

**Affiliations:** 1 Department of Pathology, University of Health Sciences, Lahore, Punjab, Pakistan; 2 Department of Morbid Anatomy and Histopathology, University of Health Sciences, Lahore, Punjab, Pakistan

**Keywords:** Carcinoma, Renal Cell, Ki-67 Antigen, Minichromosome Maintenance Complex Component 2, Cell Proliferation

## Abstract

**Introduction/Background:**

Fuhrman nuclear grade is the most important histological parameter to predict prognosis in a patient of renal cell carcinoma (RCC). However, it suffers from inter-observer and intra-observer variation giving rise to need of a parameter that not only correlates with nuclear grade but is also objective and reproducible. Proliferation is the measure of aggressiveness of a tumour and it is strongly correlated with Fuhrman nuclear grade, clinical survival and recurrence in RCC. Ki-67 is conventionally used to assess proliferation. Mini-chromosome maintenance 2 (MCM-2) is a lesser known marker of proliferation and identifies a greater proliferation faction. This study was designed to assess the prognostic significance of MCM-2 by comparing it with Fuhrman nuclear grade and Ki-67.

**Material and Methods:**

n=50 cases of various ages, stages, histological subtypes and grades of RCC were selected for this study. Immunohistochemical staining using Ki-67(MIB-1, Mouse monoclonal antibody, Dako) and MCM-2 (Mouse monoclonal antibody, Thermo) was performed on the paraffin embedded blocks in the department of Morbid anatomy and Histopathology, University of Health Sciences, Lahore. Labeling indices (LI) were determined by two pathologists independently using quantitative and semi-quantitative analysis. Statistical analysis was carried out using SPSS 20.0. Kruskall-Wallis test was used to determine a correlation of proliferation markers with grade, and Pearson's correlate was used to determine correlation between the two proliferation markers.

**Results:**

Labeling index of MCM-2 (median=24.29%) was found to be much higher than Ki-67(median=13.05%). Both markers were significantly related with grade (p=0.00; Kruskall-Wallis test). LI of MCM-2 was found to correlate significantly with LI of Ki-67(r=0.0934;p=0.01 with Pearson's correlate). Results of semi-quantitative analysis correlated well with quantitative analysis.

**Conclusion:**

Both Ki-67 and MCM-2 are markers of proliferation which are closely linked to grade. Therefore, they can act as surrogate markers for grade in a manner that is more objective and reproducible.

## INTRODUCTION

Renal cell carcinoma (RCC) is the most common renal tumor accounting for 3% of cancer deaths in males (1). Fuhrman nuclear grading is the most important histological parameter to predict prognosis of the patients. Over the years Fuhrman nuclear grading has come to be accepted by most pathologists in the World. However, many studies have shown that it suffers from substantial intra-observer and inter-observer variation. Fuhrman nuclear grade relies on three factors for its categorization: the nuclear size, nuclear pleomorphism and the nucleolar prominence. However, there is no clear guideline in cases that do not fit in any category. Most pathologists rely upon nucleolar prominence as the sole criteria for grading the tumor. The problem arises between grade II and III where pathologists have to decide whether nucleoli are large and prominent enough at low power (100X) to classify the tumor as grade III (2).

Recent evidence has suggested that Fuhrman nuclear grading has no prognostic importance in chromophobe renal tumors (3), while in papillary renal cell carcinoma only nuclear pleomorphism has any prognostic significance (4).

ISUP (International Society of Urological Pathology) published its recommendations in a meeting in 2013, where it recognized all the short comings of Fuhrman nuclear grading and proposed a new classification based on nucleolar prominence alone. However, the system does not apply to chromophobe renal carcinomas. The distinction between grade II and grade III tumors still remains subjective (5).

Therefore, there is a need for a prognostic marker that might act as surrogate for nuclear grade and be also more objective in its interpretation. Proliferation index of renal cell carcinoma as determined by Ki-67 is known to be of prognostic importance in univariate and multi-variate analysis and is known to correlate with tumor grade. Discovered by Gerdes, Ki-67 is a non-histone protein that is present in all the phases of cell cycle (G1, S, G2 and mitosis) while it is absent in non-dividing cells (G0) (6). This property makes it an excellent marker for determining the proliferation fraction of cells in tumors. Instead of relying on multiple parameters, Ki-67 requires only the presence or absence of nuclear staining as its sole criteria for interpretation. Several studies have determined a cut off value over which the expression of this marker is related with poor prognosis.

Minichromosome maintenance proteins were first reported by Maine in 1984. In an attempt to identify factors that originate DNA replication, Maine and colleagues constructed mitotically stable plasmids named minichromosomes and attempted to find mutant genes which affect the maintenance of these minichromosomes (7).

Up to 10MCM proteins have been discovered so far. This is a highly conserved group of proteins present in all eukaryotes. MCM1 is a transcription factor; MCM10 is a ring shaped hexamer which physically links Helicase to DNA polymerase during DNA replication. MCM8 has a role in mitosis, while the role of MCM9 has not been elucidated completely (8).

Here we are mainly concerned with MCM2, which forms a hexameric pre-replication complex (pre-RC) with other MCM proteins 3-7, and attaches to origin recognition complex (ORC) in association with CDC6, which acts as a recruitment factor (9). MCM2 and other proteins are also direct targets of ATM (ataxia telangiectasia mutated) and ATR (ataxia telangiectasia and Rad3 related) genes, which stop DNA replication and initiate repairs (10). MCM2 encodes a protein of 890 amino acids, and is homologous with MCM3 (11).

Several studies have shown that MCM proteins remain stable during cell cycle while their amount decreases significantly during differentiation. This is because the pre-replication complex is present throughout the cell cycle. This property makes these proteins suitable as proliferation markers (12).

Alex Freeman conducted a large study on immunohistochemical expression of MCM proteins in many normal, dysplastic and malignant tissues. Proliferation index of MCM-2 was found comparable to other proliferation markers in most normal and pathological tissue samples (13). Roddins conducted the first study on the pattern of distribution of MCM-2 in normal kidney and RCC. He found that RCC showed greater expression of this marker as compared to normal tissue. A labeling index of >20% was associated with poor prognosis (14). Dudderidge et al. 2005 compared MCM-2 with Geminin and Ki-67 and concluded that MCM-2 was a better marker than Ki-67 or Geminin to predict disease-free survival. He also suggested that semi-quantitative analysis of MCM-2 can be more easily utilized in laboratories and may act as a tool to complement Fuhrman nuclear grade to assess the prognosis of the patient more objectively. However, no attempt has been made to externally validate these results or to perform a semi-quantitative analysis to verify these claims until now (15).

We have determined the proliferation indices of fifty cases of RCC using Ki-67 and MCM-2. We have correlated the proliferation index with Fuhrman nuclear grade. We have also compared the proliferation indices of different subtypes of renal cell carcinoma. We have also compared the results of our quantitative analysis with semi-quantitative analysis.

## MATERIALS AND METHODS

### 

#### Patients and archival material

Fifty cases of renal cell carcinoma were selected from Allied Hospital Faisalabad and Sheikh Zayed Hospital Lahore. Paraffin embedded blocks along with other clinical data were acquired after permission. Patient's age, gender, tumor laterality and stage of the tumor were recorded. Nephrectomy specimens obtained from recent surgeries were grossed in the Histopathology department of University of Health Sciences according to CAP guidelines. H&E slides were prepared to determine tumor subtype, Fuhrman nuclear grade and other important prognostic parameters.

#### Immunohistochemistry

Immunohistochemical staining using Ki-67(MIB-1, Mouse monoclonal antibody, Dako) and MCM-2 (Mouse monoclonal anti-body, Thermo) was performed on the paraffin embedded blocks. Tris-EDTA Buffer antigen retrieval solution was used and the solution heated in water bath for 2 hours. Dako peroxidase block, secondary antibody and streptavidin biotinylated essays were used. Section of a reactive lymph node was used as a positive control. Any amount of nuclear staining was considered positive. Any cytoplasmic staining was ignored. Labeling indices (LI) were determined by two pathologists independently.

For quantitative analysis first hot spots were determined using low power and then approximately 1000 cells were counted in 5 high power fields. Following formulae was used to obtain the labeling index: 
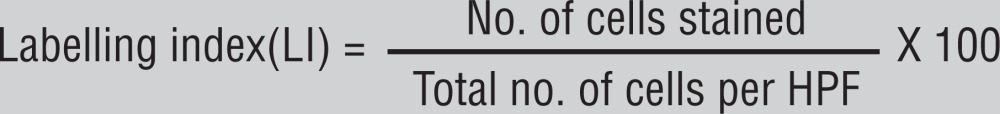



Semi-quantitative analysis was performed by analyzing different fields for highest staining and then the pathologists estimated the labeling index based on the number of cells stained in one HPF. The observers were blind to the results of semi-quantitative analysis.

### Statistical analysis

Statistical analysis was carried out using SPSS 20.0. Mean±S.D was given for quantitative variables like age, greatest dimension (T) and Labeling indices of Ki-67 and MCM-2. Kruskall-Wallis test was used to determine a correlation of proliferation markers with grade, and Pearson's correlate was used to determine correlation between both proliferation markers. Spearman's correlate rank was used to determine relationship among quantitative and semi-quantitative analysis of expression of both markers.

## RESULTS

Patients characteristics and gross and histological features Clinical and histological details are mentioned in Table-1.

**Table 1:** clinical and important histological parameters.

Total number of cases	50
Male	36
Female	14
Mean age	50.16 years
Mean of maximum size (T)	10.41cm
Grade I	8
Grade II	12
Grade III	14
Grade IV	16
Clear cell carcinoma	37
Papillary renal cell carcinoma	7
Chromophobe renal cell carcinoma	6

Expression of MCM-2 and Ki-67 and its relationship with Fuhrman nuclear grade.

**Figure 1:**
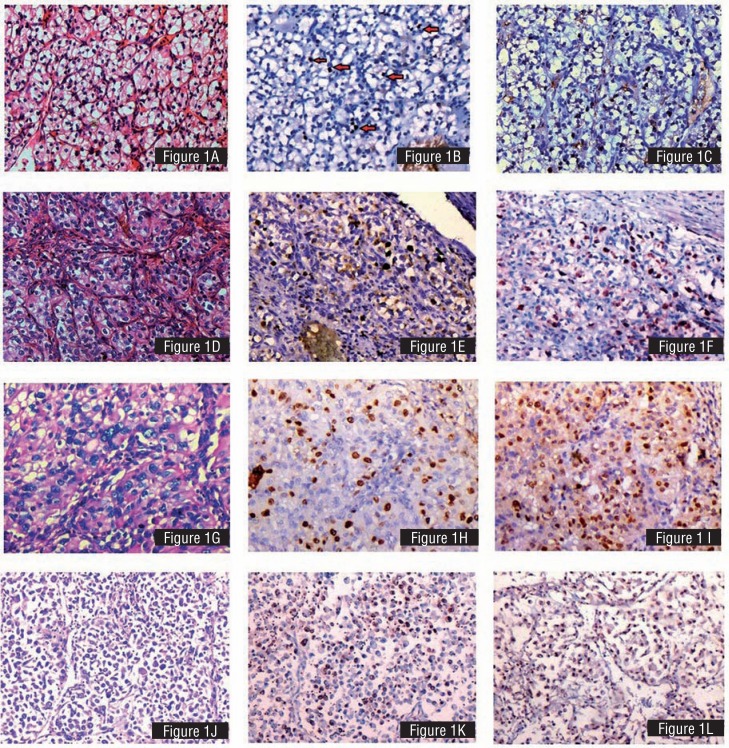
A) H&E Renal cell carcinoma grade I. Small, round and uniform nuclei. No evident nucleoli. B) Ki-67 staining in grade I renal cell carcinoma. c) MCM-2 staining in grade I renal cell carcinoma. D) Grade 2 renal cell carcinoma. Nuclei with irregular outlines and inconspicuous nucleoli. E) Ki-67 staining in grade II renal cell carcinoma. F) MCM-2 staining in grade II renal cell carcinoma. G) Fuhrman nuclear grade III, Irregular nuclei with identifiable nucleoli at 100x magnification. H) Ki-67 staining in grade III renal cell carcinoma. I) MCM-2 staining in grade III renal cell carcinoma. J) Fuhrman nuclear grade IV, Large, hyperchromatic, pleomorphic nuclei. Single or multiple nucleoli. K) Ki-67 staining in grade IV renal cell carcinoma. L) MCM-2 staining in grade IV renal cell carcinoma.

**Figure 2:**
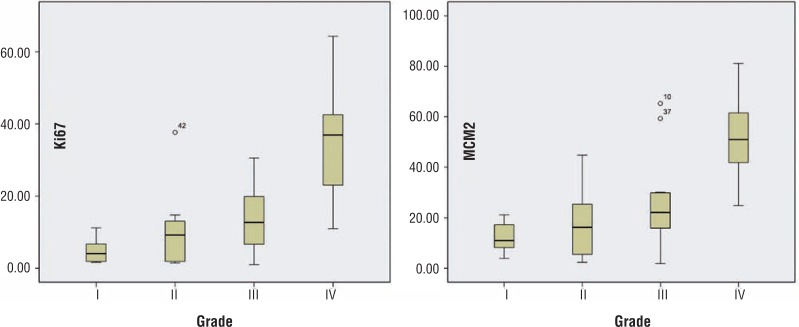
Stem and Leaf graphs showing relationship of grade with Labeling index for Ki-67 and MCM-2. Note that although there is significant overlap in the LI between grades, the mean is increasing. Black central line (median), Boxes (interquartile ranges) and ranges are enclosed by lines. The outlying cases are shown by dots.

The median proliferation index of MCM-2 and Ki-67 was 13.05% and 24.29% respectively. Labeling index of Ki-67 and MCM-2 increased with grade. For Ki-67 the LI for grade I was 4.7738±3.3% (4.05%), for grade II 9.75±10.22% (median=9.2%), for grade III 13.86±9.3% (median=12.70%) and for grade IV 35.11±14.1% (median=36.93%). MCM-2 showed increased staining in corresponding grades with LI of 12.22±5.91% (median=10.96%), 16.91±12.81% (median=16.21%), 25.18±17.97% (median=22.08%) and 52.05±15.21% (median=50.99%) for grades I through IV respectively (Figures 1 and 2, Table-2A).

**Table 2A:** Breakdown of values of proliferation index according to tumour subtype and grade. There is an increase in proliferation index in clear cell renal cell carcinoma and papillary renal cell carcinoma according to grade. No such relationship is seen in the chromophobe renal tumours. Table 2B - There is remarkable correlation between quantitative and semiquantitative analysis for both markers. In group I the median value for Ki-67 is 4.05% in quantitative analysis while it is 5% in semiquantitative analysis. MCM-2 shows 10.96% in quantitative and 10.00 in semiquantitative analysis. For grade II Ki-67(quantitative) is 9.2% versus 5% in semi-quantitative. MCM-2 shows better correlation in this grade with median values of 16.21% versus 15%. In group III Ki-67(quantitative) is 12.7% and semi-quantitative is 12.5%. MCM-2 shows (quantitative)22.08% versus 20% in semi-quantitative analysis. Finally grade IV shows Ki-67(quantitative) 36.93% versus 40% in semiquantitative. MCM-2(quantitative) 50.99% versus 55% in semiquantitative. The mean values and standard deviations also correspond with each other. Note that Ki-67(semi-quantitative) median for grade I and II is same as compared to MCM-2. Spearman's Correlate is significant in all cases except for Ki-67 grade I.

		A) Correlation of grade with labelling index according to each subtype						B) Comparison of Labelling indices of Ki-67 and MCM-2 using quantitative and semi-quantitative analysis					
		Clear cell Carcinoma		Papillary Renal cell carcinoma		Chromophobe renal cell carcinoma		Ki-67 (Quantitative)	Ki-67 (Semi-quantitative	Spearman's Correlation rank test(p)	MCM-2 (Quantitative)	MCM-2 (semi-quantitative)	Spearman's correlation rank test(p)
Grade		Ki-67	MCM-2	Ki-67	MCM2	Ki-67	MCM-2						
I	N	6	6	1	1	1	1	8	8	-0.051	8	8	0.884
	Median	4.05%	13.47%	6.67%	10.02%	1.74%	3.90%	4.06%	5.00%	p=0.904	10.97%	10.00%	p=0.004
II	N	8	8	2	2	2	2	12	12	0,88	12	12	0,876
	Median	11,10%	24,19%	5,57%	9,32%	1,67%	5,08%	9,20%	5,00%	p=0.000	16.22%	15.00%	p=0.000
III	N	9	9	2	2	3	3	14	14	0.726	14	14	0.756
	Median	14.54%	23.77%	12.27%	24.58%	2.50%	3.60%	12.70%	12.50%	p=0.003	22.09%	20.00%	p=0.002
IV	N	14	14	2	2	NA	NA	16	16	0.791	16	16	0.865
	Median	36.93%	54.60%	31.55%	41.86%	NA	NA	13.05%	10.00%	p=0.00	24.29%	20.00%	p=0.000
						Total	N	50	50		50	50	
							Median	13.05%	10.00%		24.29%	20.00%	

Proliferation indices were also determined separately for each tumor subtype. Labeling index was significantly lower for chromophobe tumors Ki-67=2.1±1.05% (median=1.83%); MCM-2=5.14±3.29% (median=4.35%) as compared to clear cell carcinomas Ki 67=21.43±16.39% (median=14.78%);MCM-2, 34.53±20.85% (median=27.55%), although no significant relationship was found between papillary Ki-67=15.06±14.25% (median=10.79%); MCM-2=25.36±19.00% (median=21.43%) and clear cell carcinomas (Table-2A).

**Figure 3:**
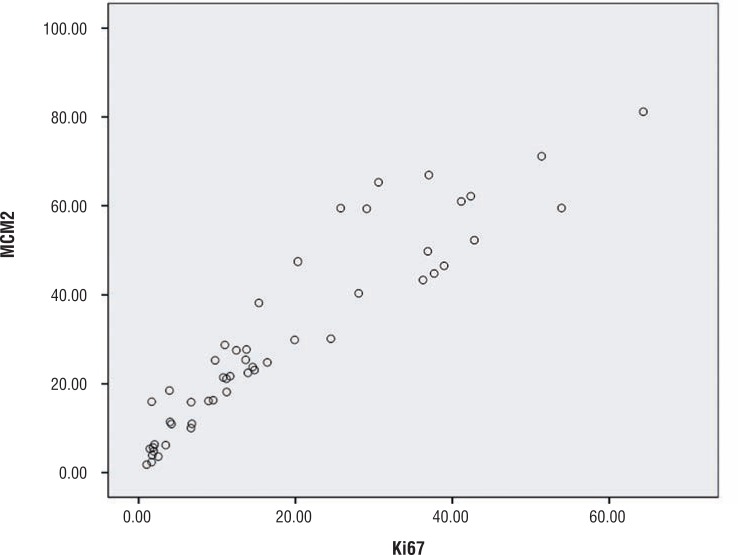
Scatter diagram showing correlation of expression between Ki-67 and MCM-2. Note that smaller increases in Ki-67 result in larger increases of MCM-2.

A correlation was sought for between ki-67 and MCM2 using Pearson's correlate and Spearman's rank test. A positive correlation of r=0.934 and ρ=0.953 was obtained and was found to be significant at p=0.01. A scatter diagram was drawn to show the correlation (Figure-3).

Semiquantitative analysis of the IHC specimen was done by estimating number of labelled cells in a single HPF in the maximum labeling area. The results produced are grouped in the Table-2B. Spearman's correlation rank test was used to determine the accuracy of semiquantitative analysis. It was ρ=0.892 in Ki-67 and ρ=0.909 in MCM-2. The correlation was significant at 0.01. However, when we split the correlation in every grade we found that Ki-67 (quantitative) versus Ki-67 (semi-quantitative) was −0.051 in grade I. The correlation was insignificant with p=0.904. In comparison MCM-2 showed a correlation of ρ=0.884. The correlation between quantitative and semi-quantitative analysis of MCM-2 was significant in every grade (Table-2B).

## DISCUSSION

Renal cell carcinoma has the poorest prognosis among all the urological tumors. Tumor stage remains the most powerful predictor of prognosis. Among histological parameters Fuhrman nuclear grade is considered the most important prognostic parameter. Our study was dominated by high grade tumors as n=30 (60%) of the tumors were either grade III or IV. This was in contrast to a study from Karachi by Latif et al. 2011 who found 66.6% grade II tumors in their study (15). Frank et al. found 46.6% tumors in either grade III or IV (16). There is very little similarity in the percentage of tumors within each grade, as it becomes difficult to fulfill all the criteria of Fuhrman nuclear grading and the pathologist comes up with his own interpretation of the system.

Papillary renal cell carcinomas are graded better using only the criteria of nucleolar prominence while Fuhrman nuclear grading seems to have no application in chromophobe renal tumors.

Ki-67 is long known to have independent prognostic importance in renal cell carcinoma, and also correlates with tumor grade. On the other hand, only a handful of studies have been previously carried out on the expression of MCM-2 in renal cell carcinoma. Ki-67 expression in our study ranged from 1.02-64.34% (median=13.05%). These results were similar to Leclercq's study (17) who recorded a range of 0-60% (median=8%) and Meierhofer et al. (2004) (18) who recorded 0-68% (median=2%). There is an overall similarity in the range of expression of Ki-67 in renal cell carcinoma. We used MIB-1 antibody in our study. However, older studies which used different versions of Ki-67 antibodies showed different results. These facts support our assumption that subjective variations have lesser role to play when assessing the expression of these immuno markers.

On the other hand, MCM-2 expression ranged from 1.80-81.80% (median=24.29%) in our study. Rodins et al. reported a range of 0.2-91.5% (median=35.7%), and Dudderidge et al. (2005) reported a range of 0.9-98.8% (median=41.6%). The expression of MCM-2 in all three studies is greater than that of Ki-67. The broader range of expression of this marker makes it relatively easier to interpret it in semi-quantitative analysis.

Our study had 6 chromophobe renal tumors. No correlation was found between their nuclear grades and proliferation index further supporting the fact that Fuhrman nuclear grade has no prognostic significance in these tumors. As a whole the proliferation index of clear cell carcinomas was greater than papillary and chromophobe renal cell carcinomas.

A good correlation was found between the proliferation indices of Ki-67 and MCM-2. MCM-2 had a greater expression than Ki-67 in nearly all the cases. Semi-quantitative analysis correlated well with the figures obtained from quantitative analysis. This suggests that objectivity of determining labeling index is largely maintained during semi-quantitative analysis and these parameters can be easily used outside of a research laboratory.

In this study we have shown that IHC expression of Ki-67 and MCM-2 not only correlates with the Fuhrman nuclear grade but is more objective and reproducible and can be used in conjunction with Fuhrman nuclear grade to determine prognosis in renal cell carcinoma.
